# Bone Cell Activity in Clinical Prostate Cancer Bone Metastasis and Its Inverse Relation to Tumor Cell Androgen Receptor Activity

**DOI:** 10.3390/ijms19041223

**Published:** 2018-04-18

**Authors:** Annika Nordstrand, Erik Bovinder Ylitalo, Elin Thysell, Emma Jernberg, Sead Crnalic, Anders Widmark, Anders Bergh, Ulf H. Lerner, Pernilla Wikström

**Affiliations:** 1 Department of Medical Biosciences, Pathology, Umea University, 901 85 Umea, Sweden; annika.nordstrand@umu.se (A.N.); erik.bovinder@umu.se (E.B.Y.); elin.thysell@umu.se (E.T.); emma.jernberg@umu.se (E.J.); anders.bergh@umu.se (A.B.); 2 Department of Surgical and Perioperative Sciences, Orthopaedics, Umea University, 901 85 Umea, Sweden; sead.crnalic@umu.se; 3 Department of Radiation Sciences, Oncology, Umea University, 901 87 Umea, Sweden; anders.widmark@umu.se; 4 Department of Molecular Periodontology, Umea University, 901 87 Umea, Sweden; ulf.lerner@umu.se; 5 Centre for Bone and Arthritis Research, Department of Internal Medicine and Clinical Nutrition at Institute for Medicine, Sahlgrenska Academy at University of Gothenburg, 413 45 Gothenburg, Sweden

**Keywords:** prostate cancer, bone, metastasis, androgen receptor, osteoblast, osteoclast, BMP

## Abstract

Advanced prostate cancer frequently metastasizes to bone and induces a mixed osteoblastic/osteolytic bone response. Standard treatment for metastatic prostate cancer is androgen-deprivation therapy (ADT) that also affects bone biology. Treatment options for patients relapsing after ADT are limited, particularly in cases where castration-resistance does not depend on androgen receptor (AR) activity. Patients with non-AR driven metastases may, however, benefit from therapies targeting the tumor microenvironment. Therefore, the current study specifically investigated bone cell activity in clinical bone metastases in relation to tumor cell AR activity, in order to gain novel insight into biological heterogeneities of possible importance for patient stratification into bone-targeting therapies. Metastasis tissue obtained from treatment-naïve (*n* = 11) and castration-resistant (*n* = 28) patients was characterized using whole-genome expression analysis followed by multivariate modeling, functional enrichment analysis, and histological evaluation. Bone cell activity was analyzed by measuring expression levels of predefined marker genes representing osteoclasts (*ACP5*, *CTSK*, *MMP9*), osteoblasts (*ALPL*, *BGLAP*, *RUNX2*) and osteocytes (*SOST*). Principal component analysis indicated a positive correlation between osteoblast and osteoclast activity and a high variability in bone cell activity between different metastases. Immunohistochemistry verified a positive correlation between runt-related transcription factor 2 (RUNX2) positive osteoblasts and tartrate-resistant acid phosphatase (TRAP, encoded by *ACP5*) positive osteoclasts lining the metastatic bone surface. No difference in bone cell activity was seen between treatment-naïve and castration-resistant patients. Importantly, bone cell activity was inversely correlated to tumor cell AR activity (measured as *AR*, *FOXA1*, *HOXB13*, *KLK2*, *KLK3*, *NKX3-1*, *STEAP2*, and *TMPRSS2* expression) and to patient serum prostate-specific antigen (PSA) levels. Functional enrichment analysis indicated high bone morphogenetic protein (BMP) signaling in metastases with high bone cell activity and low tumor cell AR activity. This was confirmed by BMP4 immunoreactivity in tumor cells of metastases with ongoing bone formation, as determined by histological evaluation of van Gieson-stained sections. In conclusion, the inverse relation observed between bone cell activity and tumor cell AR activity in prostate cancer bone metastasis may be of importance for patient response to AR and/or bone targeting therapies, but needs to be evaluated in clinical settings in relation to serum markers for bone remodeling, radiography and patient response to therapy. The importance of BMP signaling in the development of sclerotic metastasis lesions deserves further exploration.

## 1. Introduction

Bone metastatic disease is the lethal end-stage of aggressive prostate cancer [1]. For decades, almost all patients with bone-metastatic prostate cancer have been treated with androgen deprivation therapy (ADT). This reduces bone pain and temporarily retards metastatic growth, but, after some time, the disease relapses to castration-resistant prostate cancer (CRPC). New treatments for CRPC have become available in the clinic, including more efficient blockage of androgen synthesis and androgen receptor (AR) signaling, novel chemotherapies, immunotherapies, and bone-targeting treatments that all temporarily retard disease progression, although with different efficiency in different patients [2]. Recent studies have suggested the existence of molecularly diverse subtypes of prostate cancer [3,4] and our own studies have shown similar molecular diversities among bone metastases [5,6,7,8]. Thus, possibilities may exist for more individualized treatment of patients with metastatic prostate cancer than previously recognized, but therapy-predictive markers are lacking.

Bone metastases in prostate cancer patients are generally classified as osteoblastic with increased bone formation, and this is also the reason why bone scintigraphy is the preferred method to assess metastasis in those patients [9]. In contrast, other cancers such as breast, lung and renal cancer usually form osteolytic metastases with increased bone resorption [10,11,12]. This classification, however, is probably oversimplified, as prostate cancer metastases in addition to increased bone formation also show dysregulated bone resorption [10,12,13]. The skeleton in healthy individuals preserves its structural and functional integrity through constant bone remodeling. At the bone remodeling sites, bone mass is maintained by a tightly controlled balance between bone resorbing osteoclasts and bone forming osteoblasts [14]. This balance is altered when prostate cancer cells colonize the bone, resulting in increased formation of immature, less mechanically competent bone, often prone to fractures [15]. Whether a pronounced bone response is seen in all patients with metastatic prostate cancer or only in a subset of individuals with a particular molecular subtype of the disease, and whether ongoing bone remodeling influences response to different cancer treatments, are unknown.

The sclerotic phenotype of bone metastases seen in prostate cancer patients suggests a possible association between locally excessive bone formation and AR activity. Androgens stimulate AR signaling in osteoblasts, causing increased bone formation, similar to how estrogens act through estrogen receptor α (ERα) in osteoblasts [16,17]. In addition, estrogens also decrease osteoclastic life span in trabecular and endocortical bone through pro-apoptotic signaling, while androgens do not seem to have any direct effects on osteoclasts [16]. Androgens may instead be converted into estrogens and thereby indirectly inhibit bone resorption via ERα in osteoclasts [17]. Accordingly, ADT reduces bone mineral density in prostate cancer patients [18,19]. Most metastases in CRPC patients, however, maintain AR signaling despite castrate levels of testosterone in the circulation, and some also show intra-tumoral steroid levels high enough for AR activation [20,21].

We have previously studied tumor cell AR activity in prostate cancer bone metastases [7,22] and mechanisms driving AR activity in CRPC, such as intra-tumoral steroidogenesis and expression of constitutively active AR variant 7 (AR-V7) [5,6]. While most prostate cancer metastases were defined as AR-driven, some (about 20%) seemed to be non-AR-driven [7]. Patients with non-AR driven metastatic disease have few treatment options, but might benefit from therapies targeting the tumor microenvironment such as immunotherapy, bisphosphonates, receptor activator of nuclear factor kappa-Β ligand (RANKL) inhibitors, or radioisotopes [23,24]. The current study specifically investigated bone cell activity in clinical bone metastases (based on expression levels of a predefined set of osteoblast, osteoclast, and osteocyte marker genes) in relation to tumor cell AR activity [7], in order to gain novel insight into biological heterogeneities of possible importance for patient stratification into bone-targeting therapies.

## 2. Results

### 2.1. Parallel Activation of Osteoclasts and Osteoblasts in Prostate Cancer Bone Metastasis

**Figure 1:**
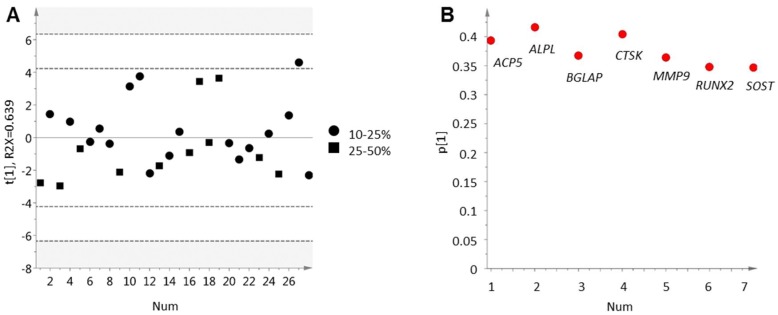
Principal component analysis of seven assigned gene products associated with bone cell activity in 28 bone metastases samples. (**A**) score plot for the first principal component. Each dot corresponds to one metastasis sample collected from prostate cancer patients. Bone content was determined within the range of 10–25% or 25–50% by histological examination of tissue sections; (**B**) loading plot showing included gene probes. Positive loading values (*p*) represent genes expressed at high levels in samples with positive score values (*t*) and vice versa.

In order to characterize ongoing bone cell activity in clinical cases of prostate cancer bone metastasis, we selected a set of genes to represent osteoclast activity (*ACP5*, *CTSK*, *MMP9*), osteoblast activity (*ALPL*, *BGLAP*, *RUNX2*) and osteocytes (*SOST*). Based on the transcript levels of these genes, a principal component analysis (PCA) model was built to capture the variation in bone cell activity among the metastatic samples. The resulting PCA model contained one significant principal component explaining 64% of the variation in the data (Figure 1A). This variation was not due to the fraction of bone tissue (10–25% or 25–50%) in the samples (Figure 1A), but was assumed to describe bone cell activity based on the chosen set of markers (Figure 1B). Therefore, the significant score vector (*t*[1]) of this model was from here on used to describe ongoing bone cell activity in the examined metastasis samples on a continuous scale.

**Figure 2:**
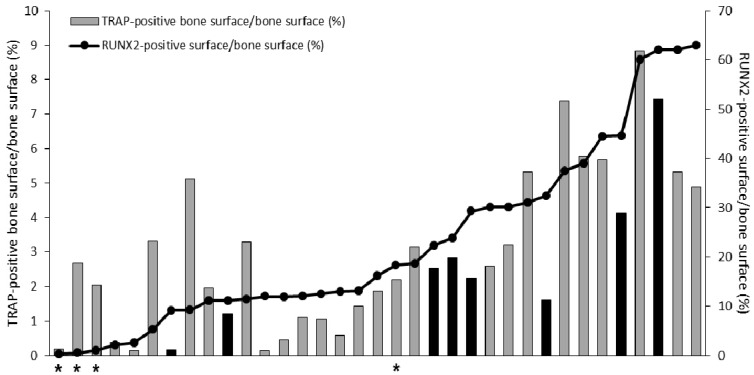
Percentage of bone surface lined by tartrate-resistant acid phosphatase (TRAP)-positive and runt-related transcription factor 2 (RUNX2)-positive cells, respectively. Each bar represents TRAP-positive bone surface in one patient sample and the corresponding dot represent the RUNX2-positive surface in the same patient. Castration-resistant prostate cancer patients are represented by grey bars and treatment-naïve patients by black bars. Patients who had undergone chemotherapy are denoted by asterisks.

**Figure 3:**
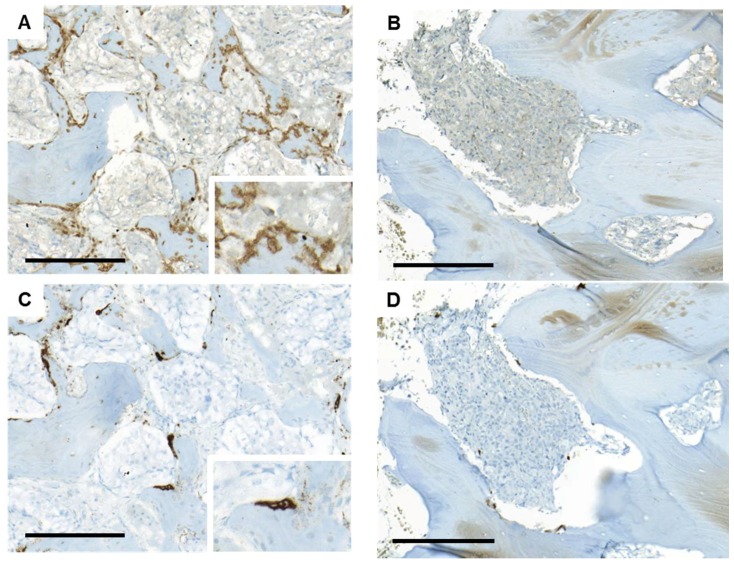

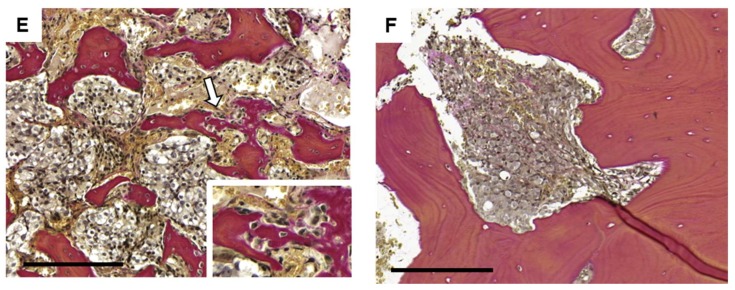
Representative immunostaining of runt-related transcription factor 2 (**A**,**B**), tartrate-resistant acid phosphatase (**C**,**D**) and van Gieson (**E**,**F**) in metastatic lesions with high (**A**,**C**,**E**) and low **(B**,**D**,**F**) bone cell activity. The area with newly formed bone is indicated with an arrow and magnified (**E**). Bar indicates 200 µm.

As can be seen in Figure 1, expression levels of the selected bone cell activity markers varied in between metastasis samples but were positively correlated within samples. Two of the markers, *RUNX2* and *ACP5* (encoding TRAP), were chosen for validation at the protein level using immunohistochemistry (Figure 2). The percentage of bone covered by RUNX2-postive cells (Figure 3A,B), likely of osteoblastic origin, and TRAP-positive osteoclasts (Figure 3C,D) was determined. The immunoreactivity scores were positively correlated to the corresponding gene expression levels for both RUNX2 (*R*s = 0.57, *p* = 0.004, *n* = 24) and TRAP (*R*s = 0.50, *p* = 0.014, *n* = 24). Importantly, the fraction of bone lined by RUNX2- and TRAP-positive cells, respectively, varied in between samples, but was significantly correlated within samples (*R*s = 0.67, *p* = 0.000009, *n* = 35) (Figure 2), supporting results from the PCA analysis and indicating parallel activation of osteoclasts and osteoblasts in prostate cancer bone metastasis. No clear difference in bone cell activity was observed between metastases from treatment-naïve and castration-resistant patients (Figure 2).

In accordance with the transcriptomic data, histological examination of van Gieson-stained tissue sections showed a large heterogeneity regarding bone cell activity between, but also within, the metastatic tissue samples. For instance, a metastatic sample could contain both areas with newly formed, osteocyte-rich woven bone (Figure 3E), and areas of old, lamellar bone (Figure 3F). The newly formed bone was most commonly located within bone marrow cavities rich in tumor cells. Only in a few cases was new bone deposited on the surface of old lamellar bone. Areas rich in RUNX2- and TRAP-positive cells were mainly found lining the surfaces of newly formed bone (Figure 3A,C). Several biopsies contained large amounts of fibrotic tissue, with or without cells, most often apart from the newly formed bone.

### 2.2. Inverse Relation between Bone Cell Activity and Tumor Cell AR Activity

To understand which clinical characteristics may differentiate patients with high respectively low bone cell activity, RUNX2 and TRAP immunoreactivity were compared to the clinical variables presented in Table 1. Serum PSA levels at metastasis surgery were found to be inversely correlated to both TRAP (*R*s = −0.51, *p* = 0.002, *n* = 34) and RUNX2 (*R*s = −0.47, *p* = 0.005, *n* = 34) immunoreactivity, indicating high bone cell activity in patients with low serum PSA levels. Notably, TRAP and RUNX2 immunoreactivity was also related to patient age at diagnosis, possibly indicating higher bone remodeling in older patients (*R*s = 0.39, *p* = 0.022, *n* = 35 and *R*s = 0.41, *p* = 0.015, *n* = 35).

Interestingly, the four patients who received chemotherapy at some point before metastasis surgery had significantly lower RUNX2 immunoreactivity than patients who had never been treated with chemotherapy (*p* = 0.015), while TRAP immunoreactivity was unaffected (Figure 2). This finding, although based on very few observations, may indicate that chemotherapy promotes a skeletal catabolic response. We found no other significant relations between immunoreactivity for RUNX2 or TRAP and the clinical variables in Table 1, although there was a tendency of less RUNX2 immunoreactivity also after radiation towards the operation site (*p* = 0.083) and after Ra-223 treatment (*p* = 0.14).

**Figure 4:**
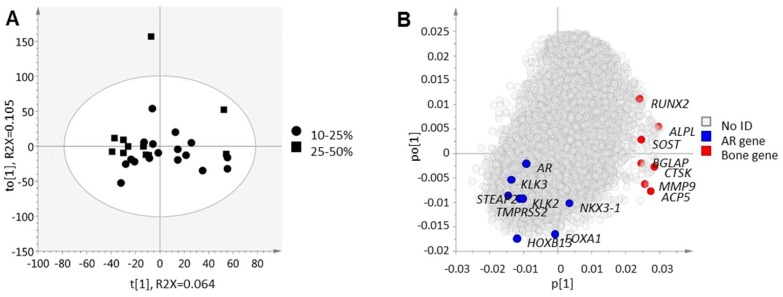
Orthogonal projections to latent structures analysis extracting the maximal variation in transcriptome data that co-vary with bone cell activity described by the weighted gene expression of *ACP5*, *ALPL*, *BGLAP*, *CTSK*, *MMP9*, *RUNX2* and *SOST t*[1] score vector. (**A**) score plot of 28 bone metastases samples where each dot corresponds to one metastasis sample collected from prostate cancer patients; (**B**) loading plot of 13847 analyzed gene products. Genes with positive loading (*p*) are highly expressed in metastases with high bone cell activity and vice versa.

For a deeper biological understanding of metastases with high bone cell activity, orthogonal projections to latent structures (OPLS) modeling was used to analyze whole-genome expression profiles of metastases in relation to the score vector in Figure 1 (Figure 4). An inverse relation was observed between bone cell activity and AR regulated genes [7], as indicated in Figure 4B for the *AR* itself, the AR co-regulators *FOXA1* and *HOXB13*, and the androgen-regulated genes *KLK2*, *KLK3*, *NKX3-1*, *STEAP2*, and *TMPRSS2*. The inverse correlation between bone cell activity and the AR associated genes was most prominent for *RUNX2*. This trend was also observed in the immunohistochemical data, where RUNX2 immunoreactivity was inversely correlated to *KLK2* (*R*s = −0.43, *p* = 0.038, *n* = 24), *KLK3* (*R*s = −0.56, *p* = 0.05, *n* = 24), and *HOXB13* (*R*s = −0.47, *p* = 0.02, *n* = 24) expression, while TRAP immunoreactivity showed no significant correlation to any of the examined AR-related genes.

Taken together, we observed here high bone cell activity in patients with low serum PSA levels and in metastases with low transcriptional AR activity.

### 2.3. Bone Formation in Prostate Cancer Metastasis Is Associated with Bone Morphogenetic Protein Signaling

To investigate what could possibly drive bone cell activity in prostate cancer bone metastases, genes with strong correlations to the score vector in Figure 1, *p* (corr) ≥ 0.5 or *p* (corr) ≤ −0.5 (Supplementary Table S1), were imported into the MetaCore software (Clarivate analytics, Philadelphia, PA, USA) for functional enrichment analysis. As anticipated, the most highly enriched process network involved genes associated with “ossification and bone remodeling” (Table 2). There was also an enrichment of networks involving epithelial-to-mesenchymal transition, cell adhesion, proliferation, bone morphogenetic protein (BMP) and growth differentiating factor (GDF) signaling, cartilage development and inflammation. No significantly enriched process was associated with genes negatively correlated to the bone cell activity score vector.

**Figure 5:**
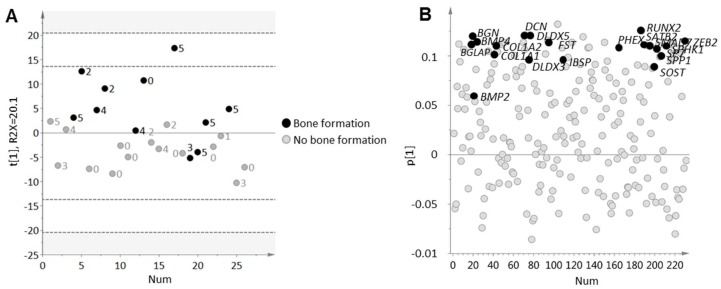
Orthogonal projections to latent structures discriminant analysis of MetaCore suggested upstream regulators of bone cells analyzed in relation to bone formation in metastasis samples. Class membership was set as ongoing or no ongoing bone formation, based on histological examination of tissue section. (**A**) score plot of 26 bone metastases samples where each dot corresponds to one metastasis sample collected from prostate cancer patients. The label (0–5) describes gradually increasing osteocyte rich tissue areas; (**B**) loading plot of 231 gene products suggested to regulate bone cell activity in metastasis samples. Genes with positive loading (*p*) are highly expressed in patient class with ongoing bone formation and vice versa. Gene products involved in bone morphogenetic protein and/or transforming growth factor β signaling are indicated.

Based on the literature of known protein interactions, the MetaCore software identified probable upstream regulators of the gene products listed in Supplementary Table S1 and, thus, of the enriched processes implied in metastases with high bone cell activity (Table 2). The suggested upstream regulators of bone cell activity (Supplementary Table S2) were then analyzed by OPLS discriminant analysis (OPLS-DA) modeling in relation to ongoing bone formation in metastasis samples (Figure 5), with the aim to identify possible osteoclast/osteoblast regulators originating from the tumor cells. Class membership was set as samples with or without active bone formation, based on histomorphometric analysis of van Gieson-stained sections (Figure 3E,F) and the criteria described under Materials and Methods. In the analysis, cases with previous chemotherapy, radiotherapy, or radiation towards operation sites were excluded and only treatment-naïve or castration-resistant patients were considered.

The resulting OPLS-DA model did not fully separate bone metastases with and without ongoing bone formation (Figure 5A), but showed a significant correlation to osteocyte content (*R*s = 0.43, *p* = 0.029) and was useful for finding gene products capturing variations in between classes. Suggested regulators with high *p* (corr) loadings indicated increased BMP signaling in cases with active bone formation (Figure 5B, Supplementary Table S3). BMP2 and BMP4 are known to stimulate osteoblast differentiation and bone formation via the bone specific transcription factors RUNX2, DLX5, and SP7 (reviewed in [25]). In addition, high levels of *DCN*, *DLX3 DLX5*, *BGN*, *ZEB2*, *FST*, and *SMAD7* indicated ongoing BMP (and/or possibly TGF-β) signaling ([26,27,28,29,30,31,32] and reviewed in [33]) in metastases with high *RUNX2*, *BGLAP*, *SATB2*, *SPHK1*, *COL1A1*, *COL1A2*, *PHEX*, *SP7*, *ALPL*, *SPP1*, *IBSP*, and *SOST* expression and detectable bone formation (Figure 5B) ([34,35] and reviewed in [33]).

**Figure 6:**
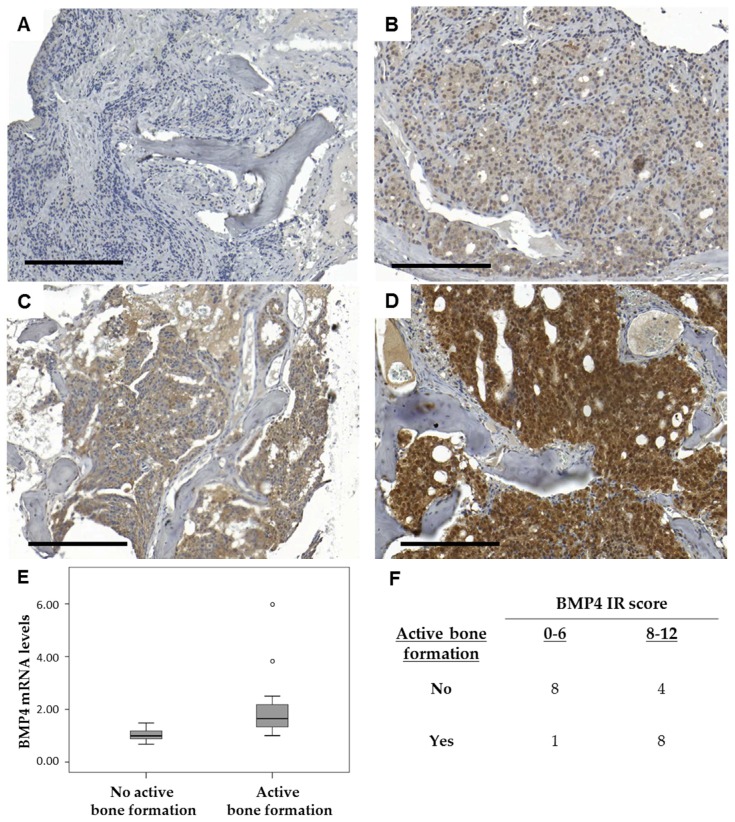
Representative sections of bone metastasis sections showing bone morphogenetic protein 4 (BMP4) immunostaining of metastatic tumor cells scored as negative (0) in (**A**); weak (1) in (**B**); moderate (2) in (**C**) and intense (3) in (**D**), with a distribution between 0 and 4 (not shown) giving immunoreactive scores (IR scores) in the range of 0–12. Bone metastases with ongoing bone formation had significantly higher BMP4 mRNA levels (**E**) and BMP4 IR scores (**F**). Bar indicates 200 µm. Circles indicate outliers and extreme values.

Immunohistochemical analysis verified specific BMP4 expression in metastatic tumor cells (Figure 6), which could be blocked by antibody incubation with an excess of BMP4 control peptide (Supplementary Figure S1). Positive BMP4 staining was occasionally detected also in endothelial cells, lipocytes, osteocytes and cells lining bone surface, but the extent of this staining showed limited variability between patients and was not further assessed. The BMP4 staining intensity of tumor cells varied between 0 and 3 (Figure 6A–D) and the distribution between 0 and 4, giving immunoreactive scores (IR scores) in the range of 0–12 (Figure 6F). In line with the *BMP4* mRNA levels (*p* = 0.001, Figure 6E), the BMP4 IR scores were significantly higher in metastases with detectable bone formation than in cases without (*p* = 0.036, *n* = 21). In other words, most cases with detectable bone formation (8 out of 9, 89%) showed high (above median) BMP4 protein expression (*p* = 0.011, *n* = 21, Figure 6F). Moreover, *BMP4* and *BMP2* mRNA levels were inversely correlated to several of the transcript levels selected to mirror AR activity in tumor cells as shown here for *BMP4; KLK3* (*R*s = −0.38, *p* = 0.019, *n* = 39), *STEAP2* (*R*s = −0.37, *p* = 0.020, *n* = 39), *FOXA1* (*R*s = −0.31, *p* = 0.053, *n* = 39), *HOXB13* (*R*s = −0.57, *p* = 0.00014, *n* = 39), and *NKX3.1* (*R*s = −0.41, *p* = 0.0092, *n* = 39). The *BMP4* and *BMP2* mRNA levels in metastasis tissue were also inversely related to serum PSA levels at metastasis surgery (*R*s = −0.35, *p* = 0.031 and *R*s = −0.56, *p* = 0.00024, *n* = 38). Taken together, our results indicate a positive relation between BMP signaling, bone cell activity, and pathologic bone formation in PC bone metastases, and those processes are in turn negatively related to AR activity in tumor cells.

## 3. Discussion

Prostate cancer bone metastases are generally classified as sclerotic, due to radiological observations of increased bone volume/density in comparison to healthy bone [9]. Nevertheless, osteolytic activity is also observed within metastatic prostate cancer patients, as judged from elevated serum levels of bone resorption markers [13,36,37]. It has been hypothesized that the sclerotic phenotype of prostate cancer may originate from intra-tumoral steroidogenesis in castration-resistant prostate cancer and thus preserved androgen levels and AR activity [16,17,21]. In the current study, variable bone cell activity was observed between samples of clinical bone metastases from prostate cancer patients and, strikingly, high osteoblast activity was found coupled to corresponding high osteoclast activity (and vice versa) within individual samples. In contrast to what could have been expected, bone cell activity was inversely correlated to transcriptional AR activity in tumor cells and to patient serum PSA levels. Ongoing bone formation was primarily observed in bone metastases with high RUNX2 expression, possibly stimulated by BMP signaling, as assessed and confirmed by robust BMP4 activity in bone forming metastases. 

Tumor cell-produced factors can influence bone remodeling, either by stimulating bone resorption or by activating osteoblasts, both processes ultimately resulting in release of factors stimulating tumor growth. This vicious cycle potentially enhances metastasis growth and aggressiveness via growth factors such as BMPs, Endothelin-1, PDGF and TGFβ [11,38]. BMPs activate specific serine-threonine kinase receptors that transmit signals by activating receptor-specific SMADs that complex with SMAD4 and translocate into the nucleus to activate transcription [39]. In complex with RUNX2 the SMADs activate transcription of genes, such as *ALPL*, *BGLAP*, *COL1A1*, *COLA1A2*, *SPP1*, *SP7*, *IBSP*, associated with osteoblastogenesis and bone formation [40]. We found *BMP4*, *BMP2* and other BMP related factors, accompanied by increased expression levels of all the above mentioned genes, to be associated with detectable, ongoing bone formation in the examined metastases. The net effect of high bone cell activity on bone formation, however, was not clarified in the current study. Evaluation of molecular evidence of bone cell activity in relation to corresponding bone scans might help in drawing conclusions, as would comparisons to serum levels of bone remodeling markers (reviewed in [41]), but such data were unfortunately not available. 

It would also be interesting to measure intra-metastatic androgen and estrogen levels in the examined cases. Androgens and estrogens are important for maintaining the balance between bone resorption and bone formation [17]. In osteoblasts, activation of the AR or the estrogen receptor α (ERα) prevents these cells from undergoing apoptosis. In mature osteoclasts, activation of the ERα promotes apoptosis and activation of ERα or AR in osteoblasts downregulates the expression of RANKL and thereby decreases osteoclastogenesis (reviewed in [42,43]). The AR, ERα, and RUNX2 also interact to modulate transcription of bone related genes (summarized in [43]). By all these mechanisms, sex hormones enhance bone mass and ADT would in general result in reduced bone formation and increased bone resorption. The observed situation in clinical bone metastases from CRPC patients, with excessive bone formation in many cases, might be explained by restored androgen levels due to local steroidogenesis and a parallel activation of osteoblast and inactivation of osteoclasts [44,45,46]. The net effect on bone formation might also depend on the presence of tumor-derived factors stimulating osteoblast differentiation and matrix formation, such as the BMPs. It is noteworthy that bone metastases with ongoing bone formation showed higher tumor cell AKR1C3 immunoreactivity [6] than cases without detectable bone formation, possibly due to intra-tumoral conversion of androstenedione to testosterone and subsequent osteoblast activation.

Besides bone formation, BMPs might be involved in a diverse range of developmental processes, including cell proliferation, differentiation, apoptosis, and angiogenesis [47,48]. BMP2 has been implicated in promoting epithelial-to-mesenchymal transition and in inhibiting tumor cell apoptosis [49]. BMP4 has been shown to promote prostate tumor growth in bone by stimulating osteogenesis [50] and, specifically, to stimulate endothelial to osteoblast conversion leading to osteoblastic bone formation in prostate cancer patients [51]. In the current study, we identified the tumor cells as the predominant source of BMP4 in metastasis with ongoing bone formation, while the cell origin of BMP2 was not examined. Another possible tumor-secreted factor found at high expression levels in metastases with ongoing bone formation was *SPP1*. This gene encodes osteopontin, which is a major non-collagenous protein in bone matrix [52]. Osteopontin facilitates the binding of osteoclasts to bone, and subsequently promotes bone resorption [53]. Over-expression of osteopontin in the LNCaP prostate cancer cell line was shown to increase proliferation and invasiveness [54] and, in a breast cancer model, osteopontin instigated the growth of otherwise quiescent metastases [55]. BMP2 signaling is known to activate *SPP1* transcription via the RUNX2-SMAD complex [40] and, accordingly, we found *SPP1* expression to be positively correlated to RUNX2 immunoreactivity in examined bone metastases. Besides BMP, the WNT signaling system has proven important for bone formation [56]. However, in the metastatic tissues examined here, we found unexpectedly low expression levels of factors involved in WNT signaling. Notably, *PTHRP* expression levels were also low in all cases examined, indicating no obvious role for this well-known osteolytic factor [57] in stimulating bone cell activity in prostate cancer bone metastasis.

We have previously identified two subgroups among CRPC bone metastases, those that have high AR and metabolic activity, but show low cellular immune responses, and those with low AR and metabolic activity, but more prominent immune responses [7]. The current study adds information to this classification of prostate cancer bone metastasis by finding high bone cell activity primarily in patients with low AR activity and ongoing inflammation (Table 2). Based on this, we hypothesize that patients with non-AR-driven bone metastases who respond poorly to standard AR inhibiting treatment may instead be specifically suited for bone- and/or immune-targeting therapies [7], while AR-driven metastases in addition to AR-targeting might benefit also from metabolic targeting [58,59], including targeting the cholesterol pathway [60,61]. Metastases with high bone cell activity may be clinically identified by relatively low serum PSA and raised levels of bone remodeling markers (see above). For a more thorough discussion on how the molecular findings of this paper could be translated into clinical practice, please see the conclusions below that further discusses limitations of the current study, but also possibilities for the future.

The use of bisphosphonates, which function to inhibit osteoclasts and osteolysis, has been proven beneficial for patients with prostate cancer bone metastases by reducing the incidence of skeletal-related events [23]. This justifies the concept of targeting bone resorption in prostate cancer patients with bone metastases. Another inhibitor of bone resorption used in the clinic is the RANKL inhibiting antibody denosumab. RANKL is mainly expressed by bone stromal cells, including osteoblasts and osteocytes, and as described above, functions as a major mediator of osteoclastogenesis and bone resorption. In the current study, expression of *TNFSF11* (RANKL*)* and its neutralizing decoy receptor *TNFRSF11B* (OPG) was undetectable in most samples. Immunohistochemical staining of RANKL and OPG might be able to determine if the ligand is present in the metastasis samples and if expression differs in relation to osteoclast activity. Another attractive therapy for prostate cancer patients with high bone remodeling may be radium-223, a short range α particle-emitting agent that acts as a calcium mimetic, meaning that it accumulates in areas of high bone turnover where it is incorporated into bone. Given the important functions of BMPs in both bone remodeling and cancer, they could also be considered attractive therapeutic targets. The natural BMP inhibitor noggin has been found to inhibit both BMP2 and BMP4, and to inhibit the expansion of PC-3 cells in vivo [62]. Dorsomorphin, a small molecule inhibitor of type I BMP receptors, has been found to inhibit ovarian cancer cell growth in vivo [63]. Notably, the present study found indications that patients undergoing chemotherapy might develop primarily osteolytic metastases, since their TRAP levels were unchanged, while their RUNX2 levels were markedly decreased. This very interesting observation deserves to be further explored in a larger cohort of patients.

## 4. Materials and Methods

### 4.1. Patient Samples

Bone metastasis samples were obtained from a series of fresh-frozen and formalin fixed paraffin embedded biopsies collected from patients with prostate cancer operated for metastatic spinal cord compression at Umea University Hospital (2003–2013). The patient series and the tissue handling have been previously described [5,7,22]. Clinical and pathological characteristics for patients included in the current study are summarized in Table 1. All patients gave their informed consent for inclusion before they participated in the study. The study was conducted in accordance with the Declaration of Helsinki, and the protocol was approved by the local ethic review board of Umea University (Dnr 03-158, 13-05-2003; Dnr 04-26M, 11-05-2007).

### 4.2. RNA Extraction and Gene Expression Analysis

Total RNA isolation and gene expression analysis of metastasis samples were previously performed, according to details given in [5,7]. Briefly, RNA was extracted from representative areas of fresh frozen bone metastases sections using the Trizol (Invitrogen, Carlsbad, CA, USA) or the AllPrep DNA/RNA/Protein Mini Kit (QIAGEN, Hilden, Germany) protocols. Nucleic acids were quantified by absorbance measurements using a spectrophotometer (ND-1000 spectrophotometer; NanoDrop Technologies, Inc., Wilmington, DE, USA). The RNA quality was analyzed with the 2100 Bioanalyzer (Agilent Technologies, Santa Clara, CA, USA) and verified to have an RNA integrity number ≥6. Gene expression array analysis was performed using the human HT12 Illumina Beadchip technique (Illumina, San Diego, CA, USA) with version 3 in [5] and version 4 in [7]. In the current study, data from the two gene expression studies were combined and samples were included if they contained a minimum of 10% bone tissue, as determined from the hematoxylin-eosin stained sections taken before RNA isolation and originally controlled to have a tumor cell content of at least 30% [5] or 50% [7]. Four patients were excluded due to previous bisphosphonate treatment. With these criteria, bone metastasis samples from 7 treatment-naïve and 21 castration-resistant patients (from originally 13 and 56 cases, respectively) were left for analysis of transcriptomic data. The bone tissue content of those metastases was further defined as 10–25% (*n* = 17) or 25–50% (*n* = 11) by visual assessment of histological sections. Beadchip data was included for all probes with average signals above two times the mean background level in at least one sample per study array. Arrays were individually normalized using the quantile method (GenomeStudio V2011.1, Illumina) and each probe was centered by the median of the samples in the corresponding dataset. Normalized datasets were merged by mapping Illumina ID and Hugo gene symbol, leaving 13,846 probes for further analysis. Redundant replication for transcripts were kept in the multivariate analysis, but removed in ontology analysis.

### 4.3. Univariate Statistics

Correlations between variables were investigated using the Spearman rank test. Groups were compared using the Mann–Whitney U test for continuous variables and the Chi square test for categorical variables. Statistical analyses were performed using the Statistical Package for the Social Sciences, SPSS 24 software (SPSS, Inc., Chicago, IL, USA).

### 4.4. Multivariate Data Analysis

Multivariate principal component analysis (PCA) was applied to create an overview of the variation in bone remodeling among metastasis samples, here based on gene expression levels of the predefined osteoblast (*ALPL*, *BGLAP*, *RUNX2*), osteoclast (*ACP5*, *CTSK*, *MMP9*) and osteocyte (*SOST*) marker genes (reviewed in [33]). The bone cell activity was represented by the first score vector (*t*[1]), capturing the largest variation in the data as a linear combination of transcript levels in the selected genes. Orthogonal projections to latent structures (OPLS) were utilized to find genes co-varying with the bone cell activity by relating the entire expression data (X) to the activity score, represented by the above *t*[1] (Y), in a linear multivariate model. In the case where OPLS was used to capture class separation, OPLS discriminant analysis (OPLS-DA) was performed. For multivariate analysis, data were centered and scaled to unit variance (UV). Sevenfold cross-validation and cross-validated analysis of variance (CV-ANOVA) testing were used to measure the statistical significance of models. Multivariate statistical analyses were performed with SIMCA software version 14.0 (Umetrics AB, Umea, Sweden).

### 4.5. Functional Enrichment Analysis

Analysis was performed using the MetaCore software (Clarivate analytics, Philadelphia, PA, USA) in order to identify enriched process networks in the data. The significance of the association between the list of molecules in the data (here gene products showing positive, *p* (corr) ≥ 0.5, or negative, *p* (corr) ≤ −0.5, correlation loadings to the score vector (*t*[1]) representative for bone cell activity in Figure 1) and process networks defined from the literature were assessed by: (i) the ratio of molecules in the data that mapped to a specific pathway in relation to the total number of molecules included in the network and (ii) the false discovery rate when applying the Fisher’s Exact test to determine the probability that the relationship between the molecules in the data set and the networks is explained by chance.

Upstream analysis was used to identify regulators with a probability to be responsible for the observed enriched process networks, based on the *p*-value for a calculated connectivity ratio between actual and expected interactions with objects in the data.

### 4.6. Immunohistochemistry and Bone Histomorphometry

Briefly, tissue sections were deparaffinized in xylene and rehydrated through graded ethanol. For histological examinations, sections were stained with hematoxylin-eosin and/or van Gieson solution. Samples with adequate tumor and bone tissue content were further immunohistochemically stained as described below.

Immunostaining for TRAP and RUNX2 was performed using the Benchmark Ultra system (Ventana, Oro Valley, AZ, USA) and the ultraView Universal DAB Detection Kit (760-500, Ventana), with antigen retrieval in the CC2 buffer and the following primary antibodies: MABF96, diluted 1:400 (Millipore, Burlington, MA, USA) and ab81357, diluted 1:100 (Abcam, Cambridge, MA, USA). BMP4 immunostaining was performed using the ab39973 antibody (Abcam, diluted 1:100) and the Envision HRP Rabbit detection system. Negative control sections were prepared by performing immunostaining procedures without adding primary antibodies and for BMP4 also by pre-incubating the primary antibody with excess (1:10 *w*/*w*) of blocking peptide (ab40140, Abcam). Immunostained sections were scanned using the Pannoramic 250 FLASH scanner and evaluated using the Pannoramic viewer 1.15.2 software (3D HISTECH, Budapest, Hungary). 

TRAP and RUNX2 immunoreactivity of cells lining bone surfaces was examined in ten randomly selected fields, with bone in close proximity to tumor cells, at 40x magnification. TRAP- and RUNX2-positive bone surface and total bone surface was measured using the ImageJ 1.50i software (NIH, Bethesda, MD, USA). A total of 35 metastases were analyzed (8 treatment-naïve and 27 castration-resistant cases), of which 24 were included in the gene expression analysis.

In corresponding van Gieson-stained sections, bone morphology was assessed by evaluating the amount of non-lamellar, newly formed, osteocyte-rich bone in close vicinity to tumor cells. The amount of such bone tissue in the biopsies was graded on a scale from 0 to 5, which reflects gradually increasing areas in relation to the total area of the biopsies. Active bone formation was evaluated as the presence or absence of cuboid osteoblasts on the surface of the newly formed bone and registered as ongoing or not ongoing.

BMP4 immunostaining was performed in 21 available samples and evaluated by scoring intensity (0 = negative; 1 = weak; 2 = moderate; 3 = intense staining) and fraction of stained cells (1 = 1–25%; 2 = 26–50%; 3 = 51–75%; 4 = 76–100%). A total IR score ranging from 0 to 12, was obtained by multiplying the staining intensity and fraction scores. 

## 5. Conclusions

This study presents evidence of a variable bone cell activity in patients with metastatic prostate cancer. Specifically, high osteoblast and osteoclast activity was observed in non-AR driven bone metastases in patients with low serum PSA levels, while low bone cell activity was seen in AR-driven metastases in patients with high serum PSA levels. This might be important to consider when stratifying patients into AR- and/or bone-targeting treatments for metastatic prostate cancer. Limitations of the study include the relatively low number of samples analyzed, the examination of only one metastasis sample per patient, and the lack of matched radiography data and serum samples for circulating biomarker analysis. Thus, the value of the molecular findings in the current study needs to be verified in well controlled clinical studies where bone metastasis samples (optimally sampled from several sites) are collected in parallel with radiography data and serum samples prior to treatment with bone-targeting therapies. Such studies will enable evaluation of bone cell activity within metastasis tissue in relation to circulating markers for bone remodeling and to the osteolytic/sclerotic metastasis phenotype as well as to patient response to therapy. If circulating markers for bone remodeling are found to correlate to bone cell activity within metastasis tissue, they will be the therapy-predictive markers of choice as bone metastasis samples are seldom sampled within clinical routine. Furthermore, the high BMP levels (specifically BMP4) found in bone metastases with signs of pathologic bone formation justify functional evaluation of BMP signaling as a suitable therapeutic target in sclerotic bone metastases.

## Figures and Tables

**Table 1:** Clinical characteristics of patients with prostate cancer who underwent surgery for metastatic spinal cord compression and where metastasis biopsies were examined by whole-genome expression analysis (*n* = 28) and/or histological analysis (*n* = 35).

Clinical Characteristic	Non-Treated *n* = 11	Castration-Resistant ^a^ *n* = 28
Age at diagnosis (years)	77 (74–82)	67 (63–74)
Age at metastasis surgery (years)	77 (74–82)	71 (68–79)
Serum PSA at diagnosis (ng/mL)	170 (60–980)	97 (37–260)
Serum PSA at metastasis surgery (ng/mL)	170 (60–980)	260 (61–490)
Gleason score at diagnosis		
7	1 (9)	9 (32)
8–10	1 (9)	14 (50)
Not available	9 (82)	5 (18)
Bicalutamide prior to surgery		
Yes		15 (54)
No		12 (43)
Not available		1 (3.6)
Chemotherapy prior to surgery ^b^		
Yes		4 (14)
No		24 (86)
Ra-223 prior to surgery		
Yes		2 (7.1)
No		26 (93)
Radiation prior to surgery ^c^		
Yes		7 (25)
No		21 (75)
Follow up after first ADT (months)	37 (24–72)	43 (25–70)
Follow up after metastasis surgery (months)	37 (24–72)	8.5 (2.2–18)

Continuous values are given as median (25th–75th percentiles) and categorical values are given as numbers (precentages). ^a^ Castration-resistant patients had disease progression after long-term androgen deprivation therapy (ADT) including surgical ablation, luteinizing hormone releasing hormone agonist therapy, and therapy with anti-androgens (bicalutamide); ^b^ Chemotherapy included taxotere in three cases and estramustine in one case; ^c^ Radiation towards operation site.

**Table 2:** The top 10 enriched process networks positively associated with bone cell activity ^a^ in prostate cancer bone metastasis.

Network	Total Gene Products	*p* (FDR ^b^)	Gene Products in Data
Development: Ossification and bone remodeling	157	3.4 × 10^−11^	*ALPL*, *FST*, *FOXC2*, *TEAD4*, *LEF1*, *PHEX*, *MEPE*, *OMD*, *DLX5*, *FOXC1*, *SP7*, *SOST*, *COL1A1*, *COL1A2*, *BGLAP*, *DLX3*, *CEBPB*, *SMAD7*, *RUNX2*, *CEBPD*, *SPP1*, *IBSP*, *BMP4*
Development: Regulation of epithelial-to-mesenchymal transition (EMT)	225	5.4 × 10^−6^	*SNAI2*, *MMP9*, *FOXC2*, *LEF1*, *PDGFD*, *COL1A2*, *ILK*, *NFKBIA*, *EDNRA*, *COL1A1*, *COL1A2*, *ACTB*, *JAK2*, *BCL2*, *VIM*, *ZEB2*, *SMAD7*, *PIK3R1*, *BMP4*
Cell adhesion: Integrin mediated cell-matrix adhesion	214	7.9 × 10^−6^	*ACTN1*, *ITGA10*, *RAPH1*, *OMD*, *ARHGEF6*, *ILK*, *TNC*, *COL1A1*, *COL1A2*, *ACTB*, *ITGA2*, *LEF1*, *PIK3R1*, *RND3*, *SPP1*, *IBSP*, *TNS3*
Cell adhesion: Cadherins	180	2.8 × 10^−5^	*ACTN1*, *FXYD5*, *ILK*, *MTSS1*, *WISP1*, *SWAP70*, *LEF1*, *DKK1*, *ACTB*, *CDH15*, *PIK3R1*, *PCDH18*, *WIF1*
Proliferation: Positive regulation cell proliferation	221	6.2 × 10^−5^	*RUNX3*, *TCIRG1*, *ZFP36L2*, *CSF1R*, *PLGF*, *GPC4*, *ILK*, *KIT*, *SCGF*, *CSPG4*, *EDNRA*, *JAK2*, *SIPR3*, *EMP1*, *GPC4*, *PIK3R1*
Signal Transduction: Bone morphogenic protein (BMP) and growth differentiation factor (GDF) signaling	91	6.2 × 10^−5^	*FST*, *ID1*, *SOST*, *BGLAP*, *PPP2R2B*, *SMAD7*, *RUNX2*, *SPP1*, *IBSP*, *BMP4*
Cell adhesion: Cell junctions	162	9.3 × 10^−5^	*ACTN1*, *ACTB*, *VIM*, *CLDN11*, *LEF1*, *ZEB2*, *CDH15*, *PIK3R1*, *YWHAH*
Inflammation: IL-6 signaling	119	9.3 × 10^−5^	*PIK3R1*, *NFKBIA*, *JAK2*, *BCL2*, *CEBPB*, *YWHAH*
Development: Cartilage development	66	1.3 × 10^−4^	*COL1A1*, *RUNX3*, *CHAD*, *COL1A2*, *SMAD7*, *RUNX2*, *BMP4*
Inflammation: Protein C signaling	108	1.6 × 10^−4^	*ACTN1*, *SPHK1*, *NFKBIA*, *ACTB*, *S1PR3*, *PROS1*

^a^ According to score vector *t*[1] in Figure 1. ^b^ FDR: False discovery rate.

## Supplementary Materials

Supplementary materials can be found at http://www.mdpi.com/1422-0067/19/4/1223/s1.
