# Supplementary figures and images for: Bone Cell Activity in Clinical Prostate Cancer Bone Metastasis and Its Inverse Relation to Tumor Cell Androgen Receptor Activity
Source: Int J Mol Sci. 2018 Apr 18;19(4):1223. doi: 10.3390/ijms19041223 (PMC5979457; doi:10.3390/ijms19041223)

Suppl. Fig. 1

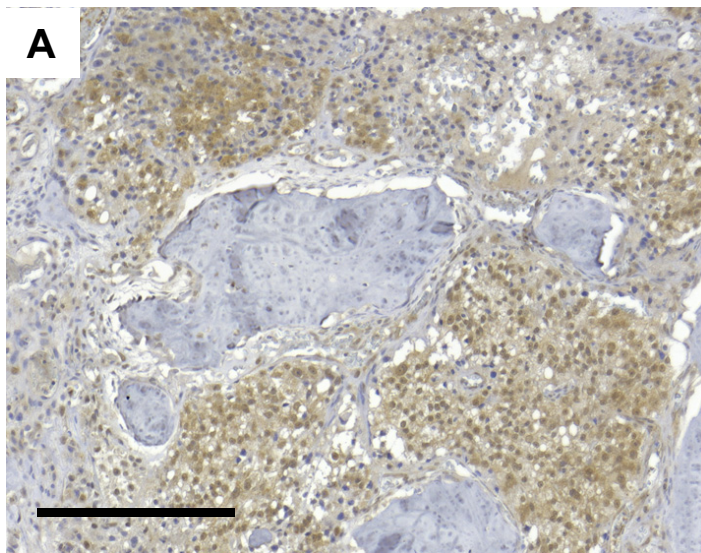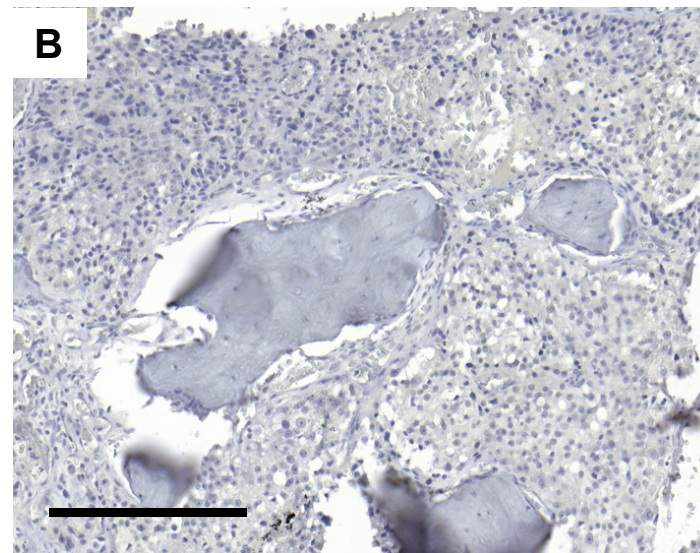

Supplement: Supplementary file 1 [file ijms-19-01223-s001.zip › suppl_FigureS1.pdf]
